# Supplementary material for: MetaRibo-Seq measures translation in microbiomes
Source: Nat Commun. 2020 Jun 29;11:3268. doi: 10.1038/s41467-020-17081-z (PMC7324362; doi:10.1038/s41467-020-17081-z)
Supplement: Supplementary file 10 — Supplementary Data 7 [file 41467_2020_17081_MOESM10_ESM.zip › File2/Confidence_High_Taxonomy/371077_out.krona.html]

Javascript must be enabled to view this page.

members
magnitude
magnitudeUnassigned
count
unassigned
taxon
rank

371077\_out

7

2
7
superkingdom

1239
phylum
7

class
7
186801

order
7
186802

541000
4
family

1263
genus
4

2
species

SRS050422\_contig\_number\_contig-100\_1195.168749SRS143991\_contig\_number\_contig-100\_8854.101092
2293190

2
species
657323

SRS019068\_contig\_number\_7254SRS063040\_contig\_number\_3703

3
family
186803

3
genus
572511

species
1

SRS077454\_contig\_number\_contig-100\_528.40724
1226324

1
species

SRS1041091\_contig\_number\_20431
765821

species
1

SRS1041136\_contig\_number\_contig-100\_1341.1342
1520805
